# Supplementary figures and images for: Differential modulation of polycomb-associated histone marks by cBAF, pBAF, and gBAF complexes
Source: Life Sci Alliance. 2024 Aug 29;7(11):e202402715. doi: 10.26508/lsa.202402715 (PMC11361369; doi:10.26508/lsa.202402715)

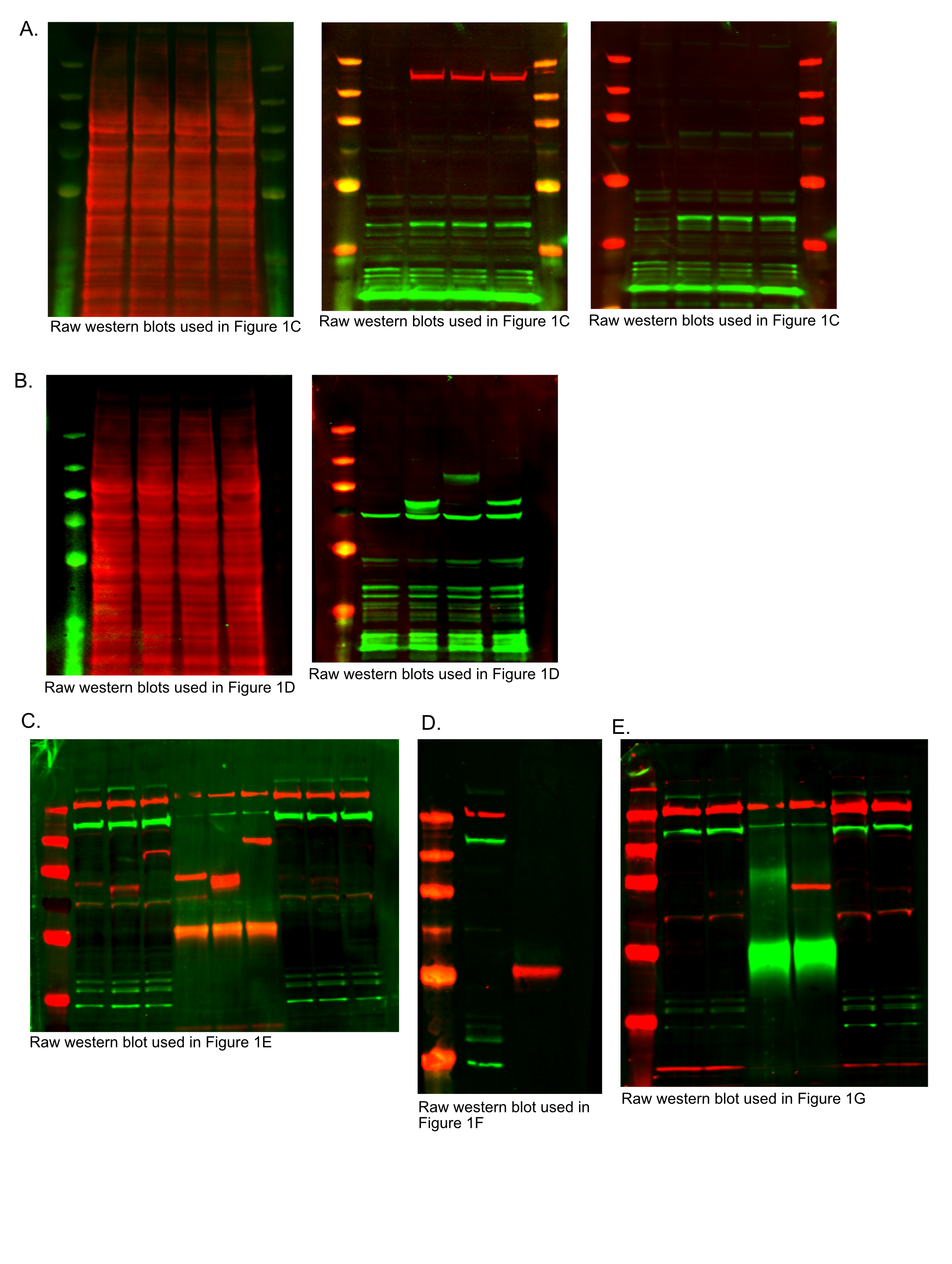

Supplement: Supplementary file 1 [file LSA-2024-02715_SdataF1.tif]

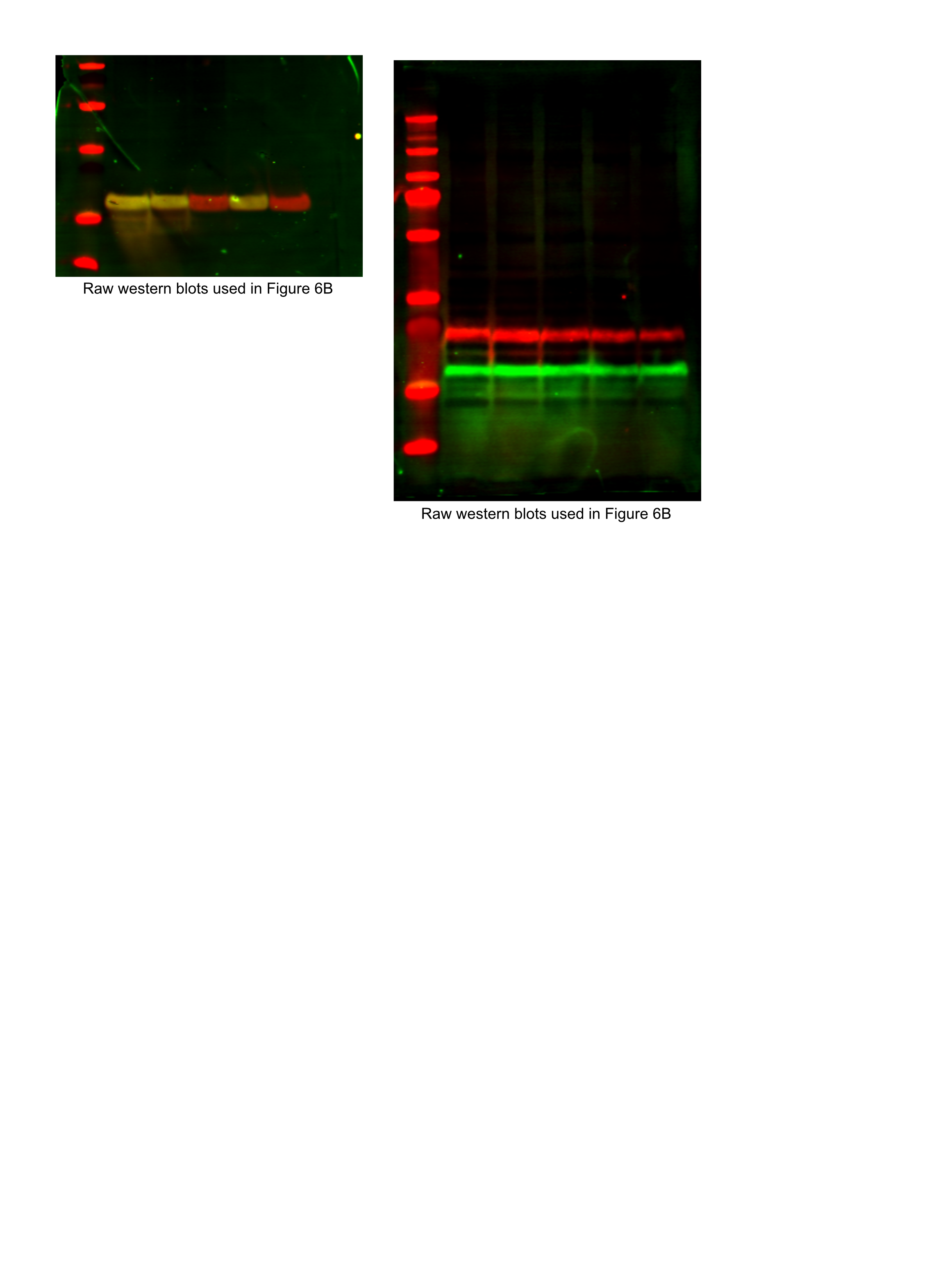

Supplement: Supplementary file 2 [file LSA-2024-02715_SdataF6.tif]
